# Venetoclax Combined with Hypomethylating Agents for Treatment-Naïve B/Myeloid Mixed Phenotype Acute Leukemia

**DOI:** 10.1155/2021/6661109

**Published:** 2021-01-12

**Authors:** Di Wu, Wenlan Chen, Zhichao Chen, Qiubai Li

**Affiliations:** Institute of Hematology, Union Hospital, Tongji Medical College, Huazhong University of Science and Technology, Wuhan 430022, Hubei, China

## Abstract

Mixed phenotype acute leukemia (MPAL) is a rare hematological malignancy that lacks consensus on optimal management. We report for the first time two cases of treatment-naïve B/myeloid MPAL patients treated with a novel chemo-free regimen using venetoclax combined with hypomethylating agents, which successfully induced complete remission with tolerable toxicities.

## 1. Introduction

Mixed phenotype acute leukemia (MPAL) is a heterogeneous group of rare acute leukemias defined as those with coexpression of myeloid and lymphoid antigens on the same blasts or those that have two separate populations of cells expressing distinct lineage characteristics, accounting for no more than 5% of all acute leukemias [1]. Due to the rarity of MPAL and the limited understanding of its pathophysiology, there is no consensus regarding optimal management. Its prognosis remains dismal, with the risk of death increased by 59% and 26% compared to that of acute lymphoblastic leukemia (ALL) and acute myeloid leukemia (AML), respectively [1].

Here, we present the first two cases of treatment-naïve B/myeloid MPAL successfully treated with a chemo-free regimen of venetoclax, a potent and selective small-molecule B-cell lymphoma/leukemia (BCL)-2 inhibitor, combined with a hypomethylating agent (HMA).

## 2. Case Report

### 2.1. Case #1

A 23-year-old female was admitted with one-week history of low fever and petechiae on her left lower extremity. Peripheral blood results showed hyperleukocytosis (112.73 × 10^9^/L), anemia (6.9 g/dL), and thrombocytopenia (51 × 10^9^/L). Physical examination revealed no hepatomegaly, lymphadenopathy, or splenomegaly. The Eastern Cooperative Oncology Group Performance Status (ECOG PS) was 1. Bone marrow evaluation showed myeloid blasts (27.5%) and primitive lymphocyte (59.5%) by cytomorphology analysis, with about 4% of the tumor cells positive for myeloperoxidase (MPO) staining. Immunophenotyping by flow cytometry revealed expression of B-cell antigens and myeloid antigens on two distinct populations of blast cells. To be specific, one population (61.64%) was phenotypically abnormal B-lineage blasts expressing CD19, CD34, cCD79a part, CD38 part, CD22, HLA-DR, CD33, and CD13, and the other (29.44%) was aberrant myeloid cells expressing CD33, CD15, CD64, CD11b part, CD11c dim, CD13 part, CD38, and CD45 dim. Chromosomal karyotyping revealed a 46, XX karyotype. First- and next-generation sequencing found no mutations of BCL-2, BRAF, KRAS, PHF6, and FBXW7. Detection for MLL-AF4, E2A-HLF, E2A-PBX1, SIL-TAL1, TEL-AML1, HOX11, HOX11L2, CAML-AF10, and BCR-ABL genes showed negative results. The patient was subsequently diagnosed as B/myeloid MPAL. BH3 profiling was not performed due to unavailability of this technique in our hospital.

After leukapheresis sessions, her white blood cell count reduced to 11.52 × 10^9^/L. For induction therapy, she received decitabine (DAC) (50 mg, intravenous infusion for 7 consecutive days) and venetoclax (100 mg p.o. day 1, 200 mg day 2, and 400 mg days 3–28). Complete remission with incomplete blood count recovery (CRi) was achieved after the first cycle, with 2.56% abnormal B-cell blasts (expressing CD19, CD34, TdT part, CD22, CD38 dim, HLA-DR, CD33, and cCD79a) detected in minimal residual disease (MRD) test by flow cytometry. We repeated this regimen for consolidation. However, her disease relapsed after this consolidation cycle, with primitive cells (42.0%) found in cytomorphology, with abnormal immunophenotyping of B-cell blasts (expressing CD19, CD34 part, CD33 part, HLA-DR, CD13 dim, CD10 part, CD22, CD38, and cCD79a part). Then, ALL-based therapy (idarubicin 12 mg/m^2^ days 1-3; vindesine 4 mg days 1, 8, 15, and 22; dexamethasone 15 mg days 1–28) was applied intravenously for one cycle, after which second CR was achieved with residual abnormal B-cell blasts (0.03%; expressing CD19, CD34, TdT dim, CD22, CD38, CD33, and CD79a part). After that, DAC (50 mg, days 1–7), venetoclax (100 mg day 1, 200 mg day 2, 400 mg days 3–28), dexamethasone (10 mg days 1–16, 5 mg days 17–20, and 2.5 mg days 21-22), and vindesine (4 mg days 1, 8, 15, and 22) were subsequently coadministered for the consolidation therapy, after which she remained CR, with 0.48% abnormal B-cell blasts (expressing CD19, CD22, CD38 dim, HLA-DR, CD33, CD34, and cCD79a) detected in the MRD test by flow cytometry. Treatment-related toxicities during the 4 cycles according to the National Cancer Institute Common Terminology Criteria for Adverse Events Version 5.0 [2] are summarized (Table 1). The patient has completed allogeneic peripheral blood stem cell transplantation (allo-PBSCT) in August 2019 and remains CR.

### 2.2. Case #2

A 24-year-old male patient was admitted with high fevers for 5 days, with a white blood cell count of 0.60 × 10^9^/L, hemoglobin of 8.3 g/dL, and platelets at 26 × 10^9^/L. There were no obvious abnormalities in his physical examination, and ECOG PS was 1. A subsequent morphology analysis of bone marrow cells revealed myeloid blasts (56.5%) and primitive lymphocyte (17%) with about 8% of the tumor cells positive for MPO staining, and flow cytometry analysis showed aberrant myeloid blasts (64.31%; expressing CD34, CD117, CD7, CD33 part, CD22 dim, CD38, CD13, CD64 part, and HLA-DR) and B-lymphoid blasts coupling with myeloid expression (3.39%; CD34, CD19, CD117 part, cCD79a, CD7, CD13, CD22, CD20, and CD38). Next-generation sequencing detected a mutation of PTPN11 gene (36.41%), and BCL-2 gene was negative. Analysis of chromosomal karyotyping, fused gene detection, fluorescence in situ hybridization, and IGH/TCR test showed negative results. Thus, a diagnosis of B/myeloid MPAL coupling with PTPN11 mutation was established. BH3 profiling was not performed either.

Antibiotics and supportive care were used to treat the severe pneumonia before the coming induction chemotherapy. However, the infection was hard to control and infectious shock occurred on the 28th day of hospitalization. Thus, to lower the infection-related risk, chemo-free induction therapy was initiated using venetoclax (100 mg day 1, 200 mg day 2, and 400 mg days 3–28) combined with azacitidine (AZA) (100 mg, subcutaneous injection for 7 consecutive days). When completed, there were no abnormal myeloid or lymphoid blasts in bone marrow morphology, and immunophenotyping revealed MRD negative status. Besides, the mutant PTPN11 gene became negative. He successfully received one more cycle of consolidation chemotherapy using IA regimen (idarubicin 20 mg days 1-3 and cytarabine 200 mg days 1–7) intravenously, after which allo-PBSCT was performed in December 2019 on patient request, and he remains CR now. Treatment-related toxicities during the 2 cycles are summarized (Table 1).

## 3. Discussion

Three mainstream regimens exist for MPAL patients: ALL-based, AML-based, and “hybrid” therapy (ALL plus AML). Based on retrospective data, experts recommend ALL-based rather than AML-based chemotherapy for its superiority in remission rates [3, 4], whereas there still exist contradictory research results. One study presented that overall responses of MPAL did not differ significantly to AML-type and ALL-type induction (84% vs 91%, *P* = 0.999) [5]. Another study analyzed 27 patients with MPAL and concluded that the CR rate of those treated with genome-wide methylation matched therapy (AML-directed therapy for AML-like MPAL and ALL-directed therapy for ALL-like MPAL) was significantly higher than those treated with unmatched regimen (72% vs 22%, *P* = 0.037). They highlighted that their data's consistency of ALL-based regimen's preponderance in response and survival rates with previous studies may be attributed to higher incidence of ALL-like MPAL, raising the question of ALL-like therapy's applicability in methylation-defined AML-like MPAL [6]. However, all these different research results have not been verified in multicenter, prospective clinical trials. In addition, complications of conventional chemotherapy, like fatal infections and hemorrhage, remain a threat to MPAL patients' survival [7].

Members of the BCL-2 protein family play a crucial role in maintaining cancer cells' survival through antagonizing proapoptotic proteins. Venetoclax, a potent and selective BCL-2 inhibitor, has shown clinical activity in hematological malignancies, including B-ALL, T-ALL, and AML, but venetoclax monotherapy is modest and explorations on combinational therapy become the mainstream choice [8, 9].

Aberrant DNA methylation contributes to the pathogenesis of both myeloid and lymphoid malignancies and is also discovered to possibly participate in aberrant transformation of hematopoietic stem cells in MPAL [10]. Decitabine (DAC) and AZA both act as HMA, and their performance in convincing response, outstanding survival benefit, and well-tolerated safety profile has enabled them to become first-line treatment options in elderly AML patients [11]. Several clinical researches also suggest their promising feasibility in B-ALL and T-ALL, especially decitabine in relapsed/refractory ALL, whereas data of this area are still limited [12–16]. An 81-year-old female MPAL, T/myeloid patient was treated by 4 cycles of DAC monotherapy to induce CR, first showing the efficacy and tolerability of HMA in MPAL [17].

When venetoclax and HMA were coadministered, the synergistic effect achieved an outstanding CR rate of 67% with manageable adverse events. The adverse events were mainly hematological and gastrointestinal toxicities in elderly AML patients unfit for standard chemotherapy [18]. In ALL, two reported cases of T-ALL who relapsed after HSCT were treated with venetoclax and decitabine and achieved second CR [19, 20]. Two cases of relapsed or refractory T/myeloid MPAL were reported to receive venetoclax combined with azacitidine or decitabine, both induced CR [21, 22]. In our female patient, whose lymphoid-linage blasts dominated, this combinational regimen did show efficacy at cycle 1 induction therapy, particularly for myeloid-linage aberrant cells, whereas the lymphoid blasts relapsed after the consolidation cycle. Subsequently, ALL-based re-induction was effective for those lymphoid blasts, and the later coadministration of DAC, venetoclax, dexamethasone, and vindesine for consolidation therapy was successful to prepare her for allo-PBSCT. As for our male patient who was frail to receive conventional intensive chemotherapy and whose myeloid-linage blasts accounted for a larger part, this regimen successfully achieved leukemia-free state and abnormal gene mutation was also cleared. Hematological and gastrointestinal toxicities were tolerable (Table 1).

In conclusion, BCL-2 inhibitor combined with HMA could be a potential option to treat MPAL, especially for those that are not suitable to receive standard chemotherapy, which needs to be further verified in future clinical trials.

## Figures and Tables

**Table 1:** Summary of treatment response and related toxicities.

	Case 1				Case 2	
Cycle	1	2	3	4	1	2
Treatment	DAC^a^ +VEN^b^	DAC +VEN	IVP^c^	DAC +VEN +VP^d^	AZA^e^ +VEN	IA^f^
Response	CRi^g^	Relapse	CR^h^	CR	CR	CR
Anemia†	3-4	2–4	2–4	2-3	3-4	2-3
Thrombocytopenia†	1–4	1–4	1–4	1–4	1–4	1–4
Neutropenia†	1–4	1–4	3-4	4	1–4	1–4
Gastrointestinal toxicities†	1-2	1-2	1-2	1-2	1-2	1-2

^a^DAC, decitabine; ^b^VEN, venetoclax; ^c^IVP, idarubicin, vindesine, and dexamethasone; ^d^VP, vindesine and dexamethasone; ^e^AZA, azacitidine; ^f^IA, idarubicin and cytarabine; ^g^CRi, complete remission with incomplete blood count recovery; ^h^CR, complete remission. †CTCAE V5.0: National Cancer Institute Common Terminology Criteria for Adverse Events, version 5.0. [2].

## Data Availability

The data used to support the findings of this study are available from the corresponding author on request.
